# Responses to Quadrivalent Influenza Vaccine Reveal Distinct Circulating CD4+CXCR5+ T Cell Subsets in Men Living with HIV

**DOI:** 10.1038/s41598-019-51961-9

**Published:** 2019-10-30

**Authors:** Megan E. Cole, Zainab Saeed, A. Torm Shaw, Yanping Guo, Katja Höschler, Alan Winston, Graham S. Cooke, Sarah Fidler, Graham P. Taylor, Katrina M. Pollock

**Affiliations:** 1 Section of Virology, Department of Infectious Disease, Imperial College London, London, UK; 2 St. Mary’s FACS facility, Imperial College London, London, UK; 3 Respiratory Virus Unit, Virus Reference Department, National Infection Service, Public Health England, London, UK; 4 Clinical Trials Centre, Jefferiss Wing, Imperial College Healthcare NHS Trust, London, UK; 5 National Centre for Human Retrovirology, Imperial College Healthcare NHS Trust, London, UK; 6 NIHR Imperial Biomedical Research Centre, London, UK

**Keywords:** Machine learning, Inactivated vaccines, Translational research

## Abstract

T cell help for B cells may be perturbed in people living with HIV (PLWH), even when HIV is suppressed, as evidenced by reports of suboptimal responses to influenza vaccination. We investigated cT_FH_ responses to the 2017–18 inactivated quadrivalent influenza vaccine (QIV) in men living with antiretroviral therapy (ART)-suppressed HIV infection who were treated in the early or chronic phase of infection, and control subjects. Here we show that seroprotective antibody responses in serum and oral fluid correlated with cT_FH_ activation and were equivalent in all three groups, irrespective of when ART was started. These responses were attenuated in those reporting immunisation with influenza vaccine in the preceding three years, independent of HIV infection. Measurement of influenza-specific IgG in oral fluid was closely correlated with haemagglutination inhibition titre. T-SNE and two-dimensional analysis revealed a subset of CD4^+^CXCR3^+^CXCR5^+^ cT_FH_ activated at one week after vaccination. This was distinguishable from cTFH not activated by vaccination, and a rare, effector memory CD4^+^CXCR5^hi^CD32^hi^ T cell subset. The data support the use of QIV for immunisation of PLWH, reveal distinct circulating CD4^+^CXCR5^+^ T cell subsets and demonstrate oral fluid sampling for influenza-specific IgG is an alternative to phlebotomy.

## Introduction

HIV infection remains a risk factor for hospitalization with influenza-related illness, particularly in older people living with HIV infection (PLWH) despite successful antiretroviral therapy (ART)^1,2^. PLWH are therefore recommended to receive yearly influenza vaccine, but efficacy is suboptimal^3,4^. Data from the early ART era indicate broad estimates in the relative risk reduction of symptomatic or confirmed influenza infection after vaccination^5,6^. Less is known about vaccine efficacy with the advent of modern HIV care, where HIV is treated irrespective of CD4 count and at higher nadir CD4 counts. This limits the size of the HIV reservoir and improves immune reconstitution^7–9^. It is likely that this will confer advantages for the vaccine responses of PLWH diagnosed recently, an important consideration as PLWH age and become vulnerable to age-associated immunodeficiency.

Despite changes in treatment guidance, suboptimal vaccine immunogenicity continues to be reported in PLWH^10–12^. This may be due to a deficiency in the specialized subsets of CD4^+^ T cells providing help to B cells. The function of tissue resident T-follicular helper cells and their similar counterparts in the blood, circulating T-follicular helper cells (cT_FH_), may be compromised despite suppression of HIV with ART. cT_FH_ have a predominantly central memory phenotype and fall into several different subsets^13^. The frequency of cT_FH_ expressing Inducible T cell COStimulator (ICOS) and progammed death 1 (PD-1) increases in adults at Day 7 post influenza vaccination and this correlates with the influenza-specific antibody response^14^. Memory cT_FH_ undergo oligoclonal expansion following inactivated influenza vaccine and promote the antibody secreting cell (ASC) response with the production of high avidity antibodies^15,16^.

cT_FH_ bear the chemokine receptor CXCR5, the ligand for CXCL13, which is highly expressed in the germinal centre and may serve as a biomarker of responses in vaccine studies^17^. Both CD4^+^ and CD8^+^ T cells expressing CXCR5 have been observed in the circulation of PLWH, and CD8^+^CXCR5^+^ T cells have potent activity against chronic viral infection^18^. Reduction in the frequency of cT_FH_ occurs in HIV viraemia, whilst during ART-mediated viral suppression, chronic immune activation may negatively impact cT_FH_ function, a defect that may be exacerbated by ageing^19–22^. The extent to which cT_FH_ are persistently infected with HIV when viremia is suppressed for many years is unclear, although it is known that CD4^+^ CXCR3^+^ T cells in the blood contain replication competent virus^23^. Tissue resident T-follicular helper cells are a sanctuary for persistent HIV contributing to the viral reservoir, which is not eradicated by standard HIV therapy^24^. It is likely that some cT_FH_ are persistently infected with HIV when viremia is suppressed, and this may be associated with perturbation of their function.

Work investigating the HIV reservoir has indicated circulating CD4^+^CD32^+^ T cells may be of interest in responses arising from B cell interactions such as the reaction to inactivated influenza vaccination. CD32, a Type I FC gamma receptor, is widely expressed on B cells, but its activity is less well understood in T-lymphocytes. CD32 has two activating subtypes, CD32a and CD32c, and one inhibiting, CD32b, which are involved in regulating the response and level of protection against influenza^25^. Although the finding that CD4^+^CD32^hi^ T cells are enriched for HIV proviral DNA has not been reproduced, questions regarding the source of CD32 on CD4^+^ T cells, and its relationship with B cells are raised by this work. CD32^hi^ CD4^+^ T cells express markers for naïve B cells and T cells as well as HIV co-receptors and when sorted, this fraction contains B cell and T cell doublets^26–32^. It is not known if these cells have a role in the response to inactivated influenza vaccination.

To investigate this, we studied the reaction to the inactivated 2017–18 Quadrivalent Influenza Vaccine (QIV). We compared influenzavaccine responses across three groups; individuals with suppressed HIV viremia who initiated ART in the early (E-HIV) or chronic phase of HIV infection (C-HIV), and staff who were sex-matched adult control participants; healthcare controls (HC). Individuals with early HIV infection were taking part in a cohort study (HEATHER), where they had laboratory evidence of primary HIV infection, were commenced on ART within three months of HIV diagnosis, and subsequently remained on ART^33^. Here we show that equivalent activation of a subset of cT_FH_ in response to vaccination occurred in all three participant groups, and was associated with subsequent seroprotective antibody titres. Amongst CD32^+^ T cells, activated cT_FH_ were more frequent at Day 7 post vaccination. These cells were distinct from cT_FH_ that were not activated by seasonal influenza vaccination and a rare circulating subset of effector memory CXCR5^hi^CD32^hi^CD4^+^ T cells.

## Results

### Study participants

Participants eligible for yearly seasonal influenza vaccination were enrolled into the study during the 2017–18 Northern Hemisphere influenza season. Adult healthcare control subjects (HC) (n = 14) and men living with HIV infection (n = 16), were included, of whom eight started ART during early HIV infection (E-HIV) and eight during chronic HIV infection (C-HIV). Early HIV infection was defined as those individuals diagnosed with primary HIV infection and started on ART within three months. C-HIV included all other PLWH attending for routine outpatient care. Participants were all male adults aged 18 years or over (Table 1). The median (IQR) CD4 count at the time of vaccination was 789 (665–1033) cells/μl in the E-HIV group and 609 (454–931) cells/μl in the C-HIV group. Participants with C-HIV had a lower nadir CD4 count 170 (80–410) cells/μl compared with E-HIV where it was 522 (466–734) cells/μl, and had a longer duration since achieving HIV suppression (viral load <50 RNA copies/ml); 90 (53.0–159.0) months vs. 19 (6.8–26.5) months. Previous influenza vaccination rates were comparable between the groups.

**Table 1:** Characteristics of study participants.

		Control		Early HIV		Chronic HIV	
		n	(% or IQR)	n	(% or IQR)	n	(% or IQR)
Sex	Male	14	(100.0)	8	(100.0)	8	(100.0)
Ethnicity	White British	8	(57.1)	3	(37.5)	2	(25.0)
	White Other	5	(35.7)	5	(62.5)	5	(62.5)
	Black British	1	(7.2)	0	(0.0)	1	(12.5)
Age years		37	(29–49)	48	(42–58)	50	(38–55)
CD4 count at vaccination, cells/µl		—	—	789	(665–1033)	609	(454–931)
CD8 count at vaccination, cells/µl		—	—	720	(558–1034)	686	(477–1350)
CD4:CD8 ratio		—	—	1	(0.9–1.4)	0.85	(0.6–1.2)
Nadir CD4 count		—	—	522	(466–734)	170	(80–410)
HIV viral load at vaccination <20 RNA copies/ml		—	—	8	(100.0)	8	(100.0)
Duration since HIV viral load <50 RNA copies/ml (months)		—	—	19	(6.8–26.5)	90	(53.0–159.0)
Self-reported influenza vaccination in last 3 years		9	(64.3)	4	(50.0)	4	(50.0)
Flu-like illness 2017–18		1	(7.1)	0	(0.0)	1	(12.5.0)
Ever smoker		4	(28.6)	2	(25.0)	4	(50.0)
Recreational drug use (ever)		0	(0.0)	2	(25.0)	2	(25.0)
Alcohol	None	1	(7.1)	1	(12.5)	3	(37.5)
	<5 units/week	2	(14.3)	2	(25.0)	0	(0.0)
	6–14 units/week	7	(50.0)	2	(25.0)	2	(25.0)
	>14 units/ week	4	(28.6)	3	(37.5)	3	(37.5)
Hepatitis B	surface antigen positive	NA	NA	0	(0.0)	0	(0.0)
	core antibody positive	NA	NA	1	(12.5)	3	(37.5)
Hepatitis C	IgG positive	NA	NA	0	(0.0)	1	(12.5)

### QIV induces an equivalent humoral response in men living with HIV infection on ART and male control subjects

Participants were immunized with Quadrivalent Influenza Vaccine (QIV); split virion inactivated (Sanofi Pasteur). The QIV contained 15 micrograms of haemagglutinin (HA) from each of A/Michigan/45/2015 (H1N1) pdm09 – like strain (A/H1N1), A/Hong Kong/4801/2014 (H3N2) – like strain (A/H3N2), B/Brisbane/60/2008-like strain (B/Brisbane) and B/Phuket/3073/2013-like strain (B/Phuket). Blood and gingival crevicular fluid (GCF) samples were taken at baseline prior to vaccination, at Day 7 and at Day 28 (Supplementary Fig. S1). The influenza-A and B strains in the 2017–18 QIV had been included in at least one of the seasonal influenza vaccines from the previous three years except A/Michigan/45/2015(H1N1) pdm09 (Supplementary Fig. S1). Individuals who had received the seasonal influenza vaccine in the past three years would therefore not have been exposed to the A/H1N1 vaccine antigen as part of a vaccination strategy.

**Figure 1:**
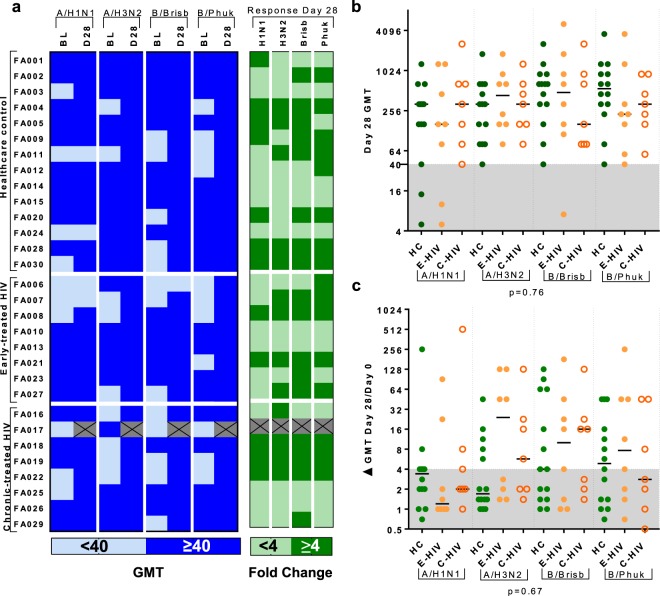
QIV induces equivalent humoral responses in PLWH and male control subjects. (**a**) Table showing haemagglutination inhibition assay (HAI) response geometric mean titre (GMT) in all participants. Left column: the baseline (BL) and Day 28 (D28) HAI titre. Those seroprotected (SP, HAI titre ≥40) are shown in dark blue, those not seroprotected (NSP, HAI titre <40) in light blue. Right column: Day 28 fold change in HAI titre. Responders (R, HAI titre ≥4 fold increase) are shown in dark green and non-responders (NR, HAI titre <4 fold increase) in light green. Crossed cells indicate incomplete data. (**b**) Graph shows Day 28 HAI titre against each of the influenza-A and B strains in, healthcare control subjects (HC), those with early-treated HIV (E-HIV) and those with chronic-treated HIV infection (C-HIV). Shaded area indicates HAI titre <40. (**c**) Graph shows Day 28 fold change in HAI titre against each of the influenza-A and -B strains in HC, E-HIV and C-HIV. Shaded area indicates HAI titre fold change <4. Lines on graph panels indicate the median. Individual data points are shown: HC, filled green circles; E-HIV, filled orange circles; C-HIV, open orange circles. p values indicate results of Kruskal-Wallis test (below graph).

Baseline antibody titres at Day 0, prior to vaccination, and humoral responses at Day 7 and Day 28 in sera were measured using the haemagglutination inhibition assay (HAI)^34^. More than half the cohort had a baseline HAI titre meeting the criteria for seroprotection (SP) for each antigen; 20/30 (66.7%) against A/H1N1, 21/30 (70.0%) against A/H3N2, 17/30 (56.7%) against B/Brisbane and 19/30 (63.3%) against B/Phuket (Fig. 1a). Following vaccination, the majority (86.2%) were seroprotected against all strains; only 2/14 (14.3%) HC and 2/8 (25.0%) E-HIV did not achieve seroprotection against A/H1N1, and only 1/8 (12.5%) E-HIV did not achieve seroprotection against B/Brisbane. Individuals were classified as Responders (R) to each antigen if they met criteria for seroconversion to the vaccine *i.e*. a ≥4-fold increase in HAI titre from baseline (Fig. 1a). There was no difference in those with E-HIV, C-HIV and HC, in the HAI titre at Day 0, 7 or 28, or the Day 28 fold change in HAI titre for any strain (Fig. 1b,c and Supplementary Fig. S2). Baseline HAI titre was higher in (Non-responders) (NR) versus R and fold change in HAI titre was higher in those not seroprotected at baseline versus those seroprotected at baseline for all strains except A/H1N1 (Supplementary Fig. S3).

### Influenza-A-specific IgG detectable in gingival crevicular fluid correlates with HAI measurements in blood and is unaffected by treated suppressed HIV infection

**Figure 2:**
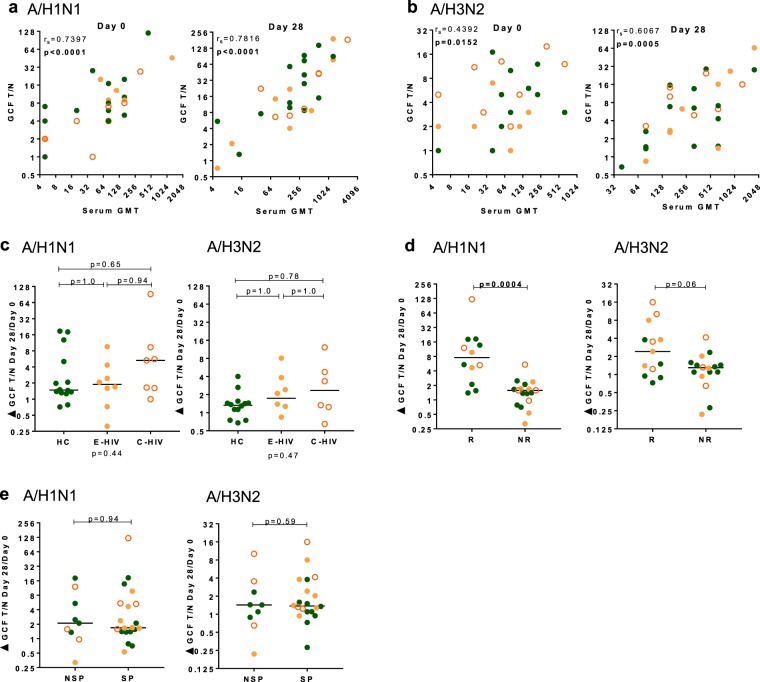
IgG response to influenza-A strains in gingival crevicular fluid correlates with serum HAI titre. (**a**) Graph panels show the correlation of serum HAI titre at Day 0 (left panel) and Day 28 (right panel) with IgG T/N ratio measured in gingival crevicular fluid (GCF) against A/H1N1. (**b**) Graph panels show the correlation of serum HAI titre at Day 0 (left panel) and Day 28 (right panel) with IgG T/N ratio measured in GCF against A/H3N2. (**c**) Graph panels show the Day 28 fold change in GCF IgG T/N against, left panel, A/H1N1, and right panel, A/H3N2 in HC, E-HIV and C-HIV. (**d**) Graph panels show the comparison of the fold change at Day 28 in GCF IgG T/N ratio in serum HAI titre responders (R) and non-responders (NR), to left panel, A/H1N1, and right panel, A/H3N2. (**e**) Graph panels show the comparison of the fold change at Day 28 in GCF IgG T/N ratio in those seroprotected (SP) and non-seroprotected (NSP) at baseline, left panel, A/H1N1, and right panel, A/H3N2. Correlations were calculated using Spearman’s rank correlation coefficient (r_s_). p values indicate results of Kruskal-Wallis test (below graph) and Dunn’s multiple comparison test (above graph) for multiple comparisons or results of the Mann-Whitney U test (above graph). Individual data points are shown on graph panels with lines drawn at the median: HC, filled green circles; E-HIV, filled orange circles; C-HIV, open orange circles.

The human gingival sulcus is 1–3 mm in depth and lies between the teeth and the vascular periodontal tissue. It is a conduit for gingival crevicular fluid (GCF), a serum exudate which contains molecules, including antibodies, and cells from the blood^35^. We hypothesised that the humoral response to QIV would be measurable in this fluid and would correlate with the serum HAI titre. Measurements of GCF IgG against influenza-A antigens were made using an ELISA assay performed on oral GCF samples taken on Day 0 and Day 28. GCF IgG against A/H1N1 correlated with the serum HAI titre from samples taken on Day 0 (p < 0.0001) and Day 28 (p < 0.0001) (Fig. 2a). GCF IgG against A/H3N2 correlated with the HAI titre from samples taken on Day 0 (p = 0.0152) and Day 28 (p = 0.0005) (Fig. 2b). Similar to our observations for serum HAI titre, neither the total GCF IgG nor its fold change differed between E-HIV, C-HIV and HC (Fig. 2c and Supplementary Fig. S4). The fold changes in GCF IgG against A/H1N1 and A/H3N2 were higher in serum responders than serum non-responders to QIV (p = 0.0004) and (p = 0.06), respectively (Fig. 2d). There was no difference in the fold change in GCF IgG for either influenza-A strain in those with or without serum seroprotection at baseline, (p = 0.94 and p = 0.59) (Fig. 2e).

### Unsupervised analysis of circulating CD4^+^ T cells reveals different responses of three phenotypically distinct CD4^+^CXCR5^+^ subsets

**Figure 3:**
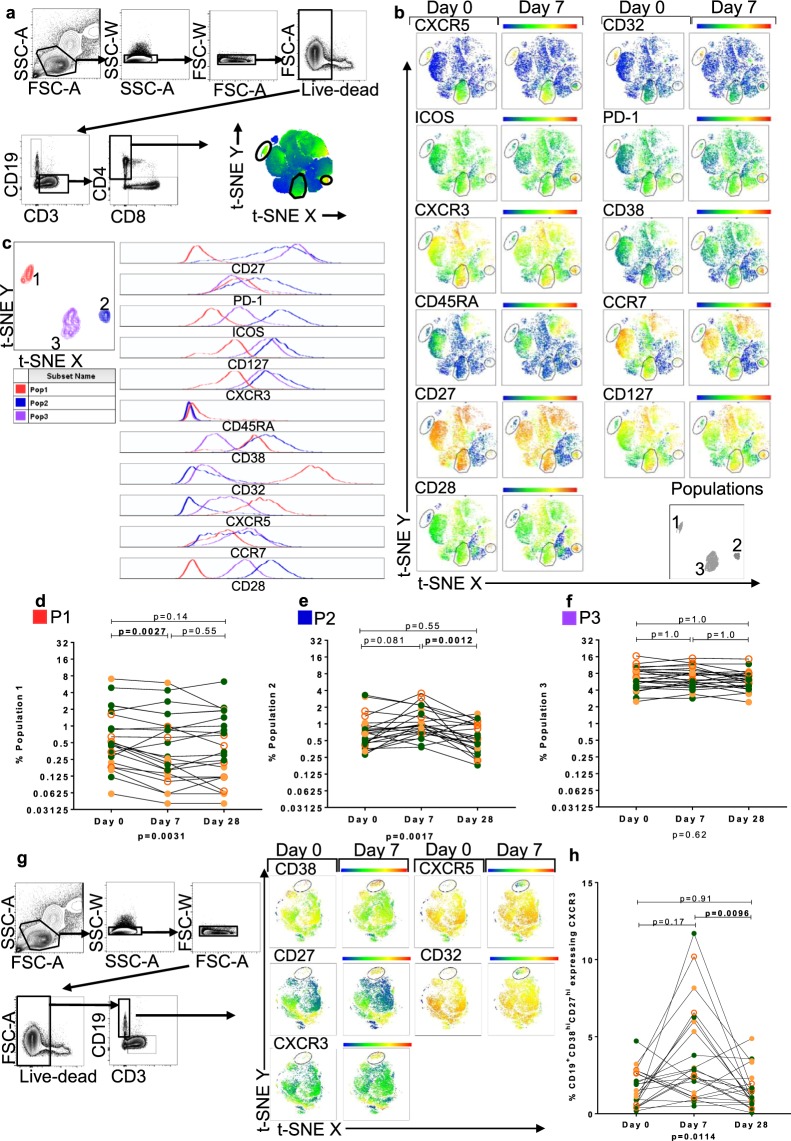
T-stochastic neighbour embedding (t-SNE) analysis identifies three CD4^+^ T cell populations expressing CXCR5 pre and post QIV. (**a**) Gating strategy and t-SNE analysis of live CD3^+^CD4^+^ lymphocytes in all participants at all time-points. Heatmaps depict the relative expression of each antigen, the scale from high (red) to low (blue) expression. (**b**) Representative t-SNE analysis of live CD3^+^CD4^+^ lymphocytes of one C-HIV participant showing the relative expression of T cell markers at Day 0 and Day 7 post QIV. Three populations of interest defined by CD32 and CXCR5 expression were gated on all participants and labelled Populations 1, 2 and 3 (bottom right panel). (**c**) Characterisation of P1–3 using histograms derived from t-SNE analysis. Data from concatenated file of all individuals’ data. Left panels show P1 (red), P2 (blue) and P3 (purple). Histograms, right panel, show the relative expression of T cell markers measured. Panels show the frequency of P1 (**d**), P2 (**e**) and P3 (**f**) from t-SNE analysis, at each time point. (**g**) Representative gating and t-SNE analysis of one C-HIV participant showing live CD19^+^ lymphocytes of all participants at Day 0 and Day 7 showing appearance of CD38^hi^CD27^hi^CXCR3^hi^ antibody secreting cells at Day 7. (**h**) Frequency of CXCR3^hi^ ASCs from t-SNE analysis at each time point. Statistical analysis was performed using a Friedman test (p value below graph) and Dunn’s multiple comparisons test (p value above graph). Individual data points are shown: HC, filled green circles; E-HIV, filled orange circles; C-HIV, open orange circles.

To investigate the CD4^+^ T cell subsets responding to QIV, we used an unsupervised computer algorithm, t-distributed stochastic neighbour embedding (t-SNE) (Fig. 3a). This resolves the high-dimensional data arising from multi-parameter FACS analysis into two dimensions, whilst preserving data structure and revealing clusters within the dataset^36^. This allowed observation of relationships in the phenotypic expression pattern of CD4^+^ T cells that might not be revealed through the use of hypothesis-driven 2-dimensional gating strategies. Heatmaps of the output were used to compare the relative expression of CD4^+^ T cell markers measured in analysis that combined all available data at each time point.

Three distinct CD4^+^ T cell populations differentiated by expression of CXCR5, were identified from all individuals both with and without HIV infection; P1, P2 and P3 (Fig. 3b). P1 was characterised by high expression of CD32 and CXCR5 and bore hallmarks for antigen experienced T_EM_, as defined by CCR7, CD45RA, CD27 and CD28 expression. Expression of CXCR3 was low in this population. Expression levels for markers of cT_FH_ activation, including inducible T cell costimulator (ICOS) and programmed death-1 (PD-1) were medium or low^13^. P2 and P3 were more similar to one another and were dissimilar from P1, with hallmarks for T_CM_, as defined by CCR7, CD45RA, CD127, CD27 and CD28 expression. CXCR3 expression was high in both P2 and P3. There were dissimilarities in expression of the activation markers PD-1, ICOS and CD38 in P2 (higher expression) and P3 (lower expression) (Fig. 3c and Table 2).

**Table 2:** Surface phenotype of CD4^+^ T cell populations identified using t-distributed stochastic neighbour embedding.

Cell cluster	Assigned colour	Expression relative to other subsets		
P1	Red	CXCR5^hi^CXCR3^lo^	CD38^mid^CD32^hi^	CD27^lo^CD127^lo^CD28^lo^
		PD-1^mid^ICOS^lo^		CD45RA^neg^ CCR7^lo/mid^
P2	Blue	CXCR5^lo/mid^ CXCR3^hi^	CD38^hi^CD32^lo/mid^	CD27^mid/hi^CD127^hi^CD28^hi^
		PD-1^hi^ICOS^hi^		CD45RA^neg^CCR7^mid^
P3	Purple	CXCR5^mid^ CXCR3^hi^	CD38^lo^CD32^lo/mid^	CD27^hi^CD127^hi^ CD28^mid/hi^
		PD-1^mid^ICOS^mid^		CD45RA^neg^CCR7^mid^

The frequency of P2 was altered following vaccination with QIV, (p = 0.0017), with an increase at Day 7 and return to baseline at Day 28 in the majority of individuals (p = 0.081 and p = 0.0012 respectively). This confirmed changes in heatmap expression density for activation markers at Day 7, including ICOS, PD-1, CD38 and CD32, visualized in P2, but not P1 and P3 (Fig. 3d–f). These CD4^+^ T cell populations were observed in individuals with and without HIV infection, and differences in their frequencies between E-HIV, C-HIV and HC did not reach statistical significance at each time point (Supplementary Fig. S5).

Using data from the same participants, t-SNE analyses were constructed for CD19^+^ B cells to compare relative expression of the B cell markers CD38, CXCR5, CD27, CD32 and CXCR3 (Fig. 3g). Induction of a CD38^hi^CD27^hi^CXCR3^hi^CXCR5^mid/lo^CX32^mid/lo^ population of CD19^+^ cells was observed at Day 7 post QIV, corresponding to the population of antibody secreting cells (ASC) identified through 2-D FACS gating analysis. ASCs were distinct from the main body of live CD19^+^ B cells in the circulation and were clearly visible in t-SNE, appearing at Day 7. These cells were more frequent at Day 7 for some but not all individuals (p = 0.17) and returned to baseline at Day 28 post QIV (p = 0.0096) (Fig. 3h).

### Hierarchical relationship of T cell populations characterised by CXCR5 expression is revealed through unsupervised analysis of the circulating T cell response to QIV

**Figure 4:**
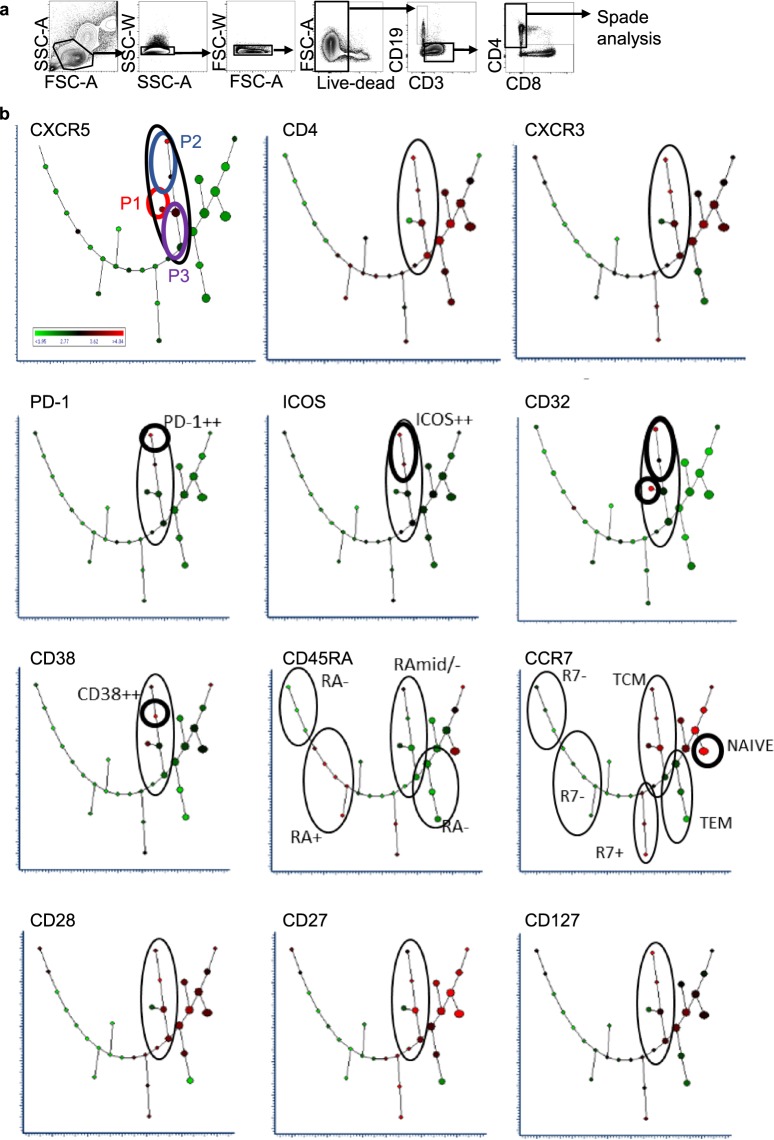
Spanning-tree Progression Analysis of Density-normalized Events (SPADE) of CD4^+^ T cells. (**a**) Representative gating strategy of live CD4^+^ T cells from one individual. (**b**) SPADE analysis of live CD3^+^CD4^+^ cells showing combined flow cytometry standard (FCS) dataset from all participants at all time-points. Cluster size indicates relative frequency of live CD3^+^CD4^+^ events. Cluster colour indicates relative marker expression; with scale from green (low) to red (high).

**Figure 5:**
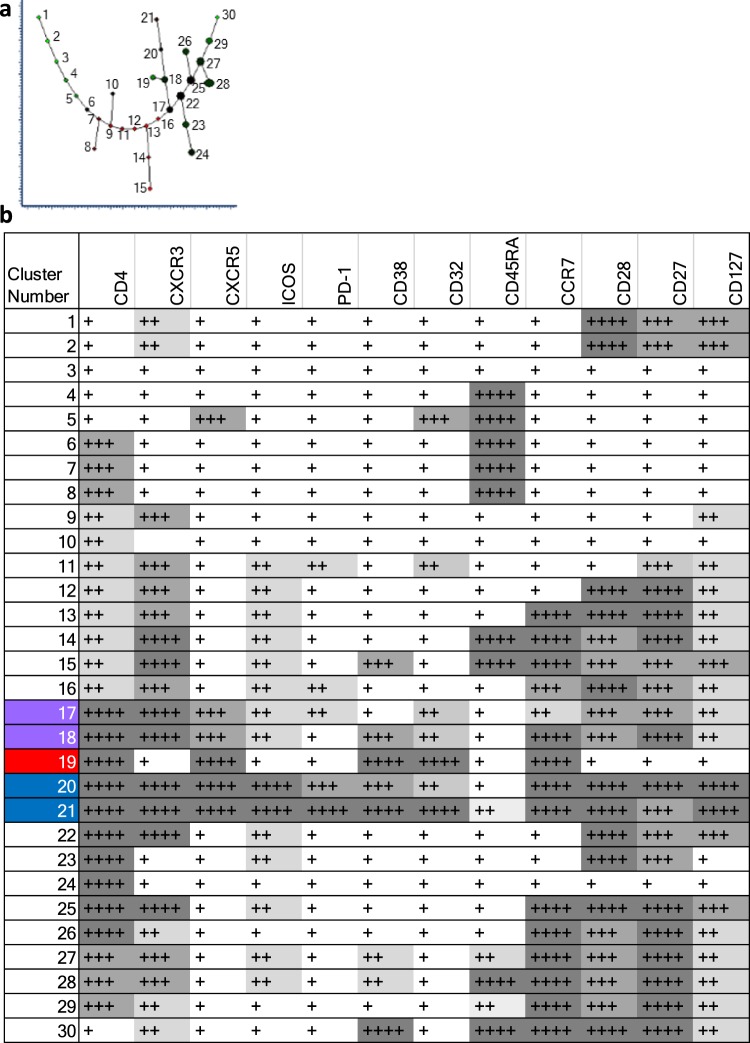
Summary of relative cellular marker expression on CD4^+^ T cell populations using Spanning-tree Progression Analysis of Density-normalized Events (SPADE). (**a**) Plot showing the assigned cluster number. (**b**) Panel shows the cluster number, left column, and relative expression of each marker denoted as low-expression (+ white background); mid-expression (++ light grey background); mid/high-expression (+++ mid-grey background), high-expression (++++ dark grey background). Translation of SPADE cluster colour into comparative expression levels was through visual discrimination on each SPADE readout using the combined dataset. Highlighted clusters in column 1 correspond to those bearing similarities to CD4^+^ T cell populations of interest identified using t-SNE; clusters 17–18 in purple (P3), cluster 19 in red (P1) and clusters 21 and 21 in blue (P2).

Spanning-tree Progression Analysis of Density-normalized Events (SPADE) was used to investigate the hierarchical relationship between the CD4^+^ T cell populations of interest identified using t-SNE. This algorithm creates 2-dimensional trees from multi-dimensional data where the cluster size is indicative of event frequency, and colour of the median expression of each selected marker. Branching between related cell clusters allows inference of hierarchy and relationship^37^. A major branch of five cell clusters (black loop) contained populations expressing CXCR5 (Fig. 4a,b). Clusters in the major branch were most closely allied to the T_CM_ or T_EM_ phenotype as evidenced by expression levels of CD45RA, CCR7, CD28 and CD27 (black loop). Amongst these were two clusters, 17 and 18, most closely related to the main population of T_CM_ (purple loop). Three clusters were downstream from cluster 18, clusters 20 and 21 (blue loop) and cluster 19 (red loop). Comparison of the expression pattern of these clusters and comparison with t-SNE analysis indicated cluster 19 (red loop) to be most similar to the CD32^hi^ P1, clusters 20 and 21 (blue loop) to the activated cT_FH_ of P2 and clusters 17 and 18 (purple loop) to the cT_FH_ of P3 (Figs 4b and 5a,b). These clusters (17–19) did not highly express cT_FH_ activation markers ICOS and PD-1. Clusters 20 and 21, were ICOS and PD-1 positive, with cluster 21 expressing these activation markers most highly. Clusters 17, 18 and 19 were present in all individuals and clusters 20 and 21 more variably (Supplementary Fig. S6a–c and Supplementary Table S1).

### Activation of circulating T-follicular helper cells correlates with fold change in HAI antibody titre following vaccination with QIV

**Figure 6:**
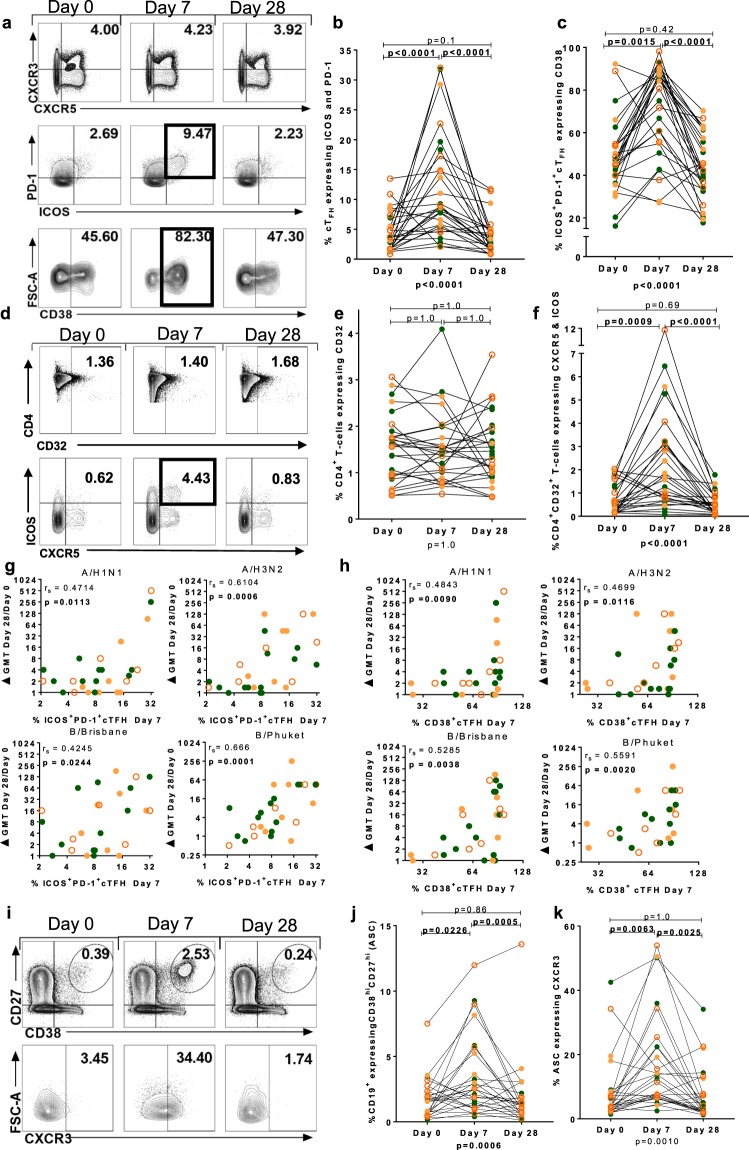
Activation of cT_FH_ and antibody secreting cells by vaccination with QIV. (**a**) Gating strategy for CD4^+^CXCR5^+^CXCR3^+^ cT_FH_. Panels show (**b**) frequency of ICOS^+^PD-1^+^ cT_FH_, (**c**) frequency of ICOS^+^PD-1^+^ cT_FH_ expressing CD38. (**d**) Gating strategy for CD4^+^CD32^+^ T cells and (**e**) frequency of CD4^+^CD32^+^ T cells across time points. (**f**) Frequency of CD4^+^ CD32^+^ CXCR5^+^ ICOS^+^ T cells at all time points. (**g**) Panels show correlation of activated cT_FH_ cells at Day 7 with fold change at Day 28 of HAI titre for A/H1N1 (top left), A/H3N2 (top right), B/Brisbane (bottom left) and B/Phuket (bottom right). (**h**) Correlation of CD38^+^cT_FH_ cells at Day 7 with fold change at Day 28 of HAI titre for A/H1N1 (top left), A/H3N2 (top right), B/Brisbane (bottom left) and B/Phuket (bottom right). (**i**) Gating strategy for CD27^hi^CD38^hi^CD19^+^ antibody secreting cells (ASC). (**j**) Frequency of CD27^hi^CD38^hi^CD19^+^ ASCs and (**k**) frequency of CXCR3^+^ASCs at each time point. Spearman’s rank correlation coefficient (r_s_) is shown. Intra-individual comparisons between time-points were performed using Friedman test; (p value below graph) and p values of the Dunn’s multiple comparisons test (above graph). Individual data points are shown on graph panels: HC, filled green circles; E-HIV, filled orange circles; C-HIV, open orange circles.

Sequential two-dimensional gating was used to compare T cell activation indicated by unsupervised analysis with humoral responses. Live CD3^+^CD4^+^CXCR5^+^CXCR3^+^ cT_FH_ were analysed (Fig. 6a and Supplementary Fig. S7). The frequency of PD-1^+^ICOS^+^cT_FH_ increased significantly at Day 7 (p < 0.0001), and then returned to baseline levels at Day 28 (p < 0.0001) (Fig. 6b). There was a significant increase in the frequency of CD38^+^PD-1^+^ICOS^+^cT_FH_ at Day 7 (p = 0.0015), that returned to baseline levels at Day 28, (p < 0.0001) (Fig. 6c). The frequency of cT_FH_ and activated cT_FH_ was not different between those with and without HIV infection, except for a slightly increased frequency of cT_FH_ at Day 7 in three individuals with C-HIV (Supplementary Fig. S8). The frequency of bulk CD4^+^ cells expressing CD32 was unchanged throughout the study (Fig. 6d,e). CD4^+^CD32^+^ T cells bearing the canonical markers of cT_FH_ (CXCR5^+^ICOS^+^) increased in frequency at Day 7 (p = 0.0009), and returned to baseline at Day 28 (p < 0.0001), in a response similar to that observed for other T cell activation markers (Fig. 6f) The phenotype and response of CD32^+^ T cells in P1-P3 was verified in 2-D gating analyses (Supplementary Fig. S9). No consistent difference in the concentration of the soluble ligand for CXCR5, CXCL13, was observed in the serum pre and post vaccination or between participant groups (Supplementary Fig. S10).

The frequency of ICOS^+^PD-1^+^cT_FH_ at Day 7 correlated positively with the fold change in HAI titre at Day 28 for all strains; A/H1N1 (p = 0.0113), A/H3N2 (p = 0.0006), B/Brisbane (p = 0.0244) and B/Phuket (p = 0.0001) (Fig. 6g). The frequency of CD38^+^ICOS^+^PD-1^+^cT_FH_ at Day 7 correlated positively with the fold change in HAI titre at Day 28 for all strains; A/H1N1 (p = 0.009), A/H3N2 (p = 0.0116), B/Brisbane (p = 0.0038) and B/Phuket (p = 0.002) (Fig. 6h).

The CD19^+^ antibody secreting cell (ASC) response following vaccination with QIV was analysed in the same samples at the same time-points as the T cell response. ASCs were CD19^+^CD38^hi^CD27^hi^ (Fig. 6i and Supplementary Fig. S11). There was no difference in the frequency of ASCs between HC, E-HIV and C-HIV at any time point (Supplementary Fig. S12). The majority of CD19^+^ cells, median (IQR) 92.22% (88.21–94.08) expressed CD32 and CXCR5 and this did not differ between individuals with and without HIV infection (Supplementary Fig. S12). ASCs expressed CXCR3 consistent with a previous report^38^. Induction of CD38^hi^CD27^hi^ ASCs was observed following vaccination with QIV at Day 7 (p = 0.0226) with resolution to baseline at Day 28 (p = 0.0005) (Fig. 6j). The frequency of ASCs expressing CXCR3 increased significantly at Day 7 (p = 0.0063) and resolved to baseline at Day 28 (p = 0.0025) (Fig. 6k). A correlation between the frequency of activated ASCs at Day 7 and the fold change in HAI titre at Day 28 was not observed for any strain (Supplementary Fig. S13).

### Circulating CD8^+^ CXCR5^+^ T-follicular cytotoxic cells are not activated by vaccination with QIV at Day 7

**Figure 7:**
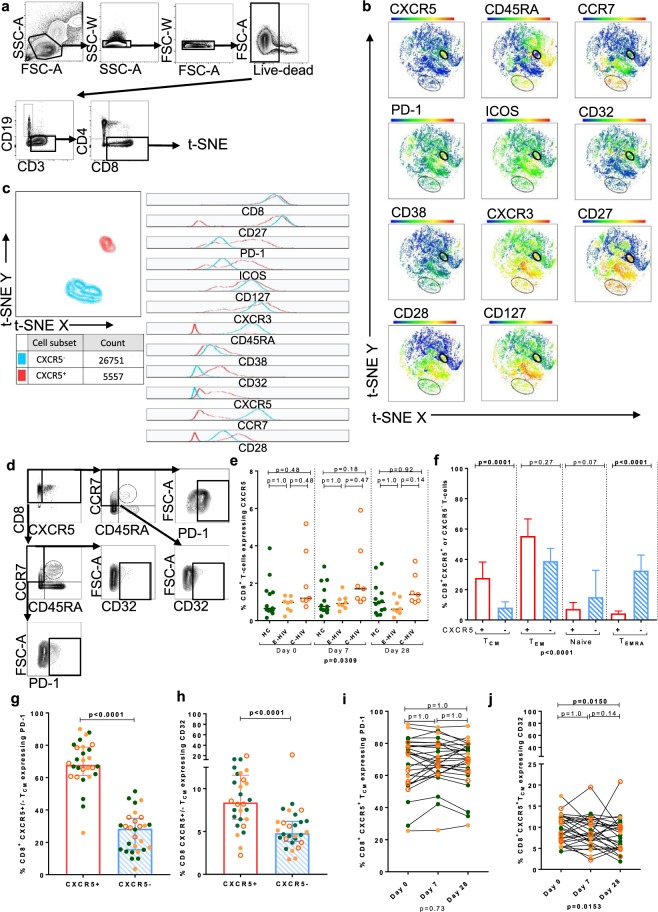
Identification of a rare population of CXCR5^+^CD8^+^ central memory T cells that is not activated by QIV. (**a**) Gating strategy and t-SNE of live CD3^+^CD8^+^ lymphocytes of all participants at all time points. Heatmaps show the relative expression of each marker. (**b**) t-SNE of live CD3^+^CD8^+^ lymphocytes of one C-HIV participant showing the CXCR5^+^ and CXCR5^−^populations. (**c**) Characterisation of CD8^+^CXCR5^−^(blue peaks) and CD8^+^CXCR5^+^ (red peaks) T cells using histograms derived from t-SNE on concatenated data from all individuals to determine the relative expression of cellular markers. (**d**) FACS gating strategy for CD8^+^CXCR5^+^ T cells and T cell memory subsets. (**e**) Comparison of the frequency of CXCR5^+^CD8^+^ T cells in HC, E-HIV, C-HIV at all time-points (**f**) Comparison of the frequency of CD8^+^CXCR5^+^ (red columns) and CD8+CXCR5^-^ (blue columns) T cells that were, from left to right, central memory cells (T_CM_: CD45RA^−^CCR7^+^), (T_EM_: CD45RA^−^CCR7^−^), (Naive: CD45RA^+^CCR7^+^), (T_EMRA_: CD45RA^+^CCR7^−^). Comparison of the frequency of CD8^+^CXCR5^+^ and CD8^+^CXCR5^−^T_CM_-cells expressing (**g**) PD-1 and (**h**) CD32. (**i**) Frequency of CD8^+^CXCR5^+^ PD-1^+^ T cells and (**j**) CD8^+^CXCR5^−^PD-1^+^ T cells at all time^−^points. P values were derived using a Kruskal-Wallis or Friedman test (p value below graph) and p values of the Dunn’s multiple comparisons test (above graph). Comparisons between groups were performed using a two-tailed Mann-Whitney U test.

T-SNE analysis of live CD8^+^ T cells, revealed a rare population expressing CXCR5 (Fig. 7a–c). FACS gating analysis of CD8^+^CXCR5^+^ T cells was undertaken to compare relative expression of memory phenotype markers and relative frequency amongst the different groups studied (Fig. 7d and Supplementary Fig. S14). CD8^+^CXCR5^+^ T cells were present at a frequency of median (IQR) 1.00% (0.58–1.49) of CD8^+^ T cells. There was a trend towards a higher frequency of these in C-HIV but this was not statistically significant different in post-test comparisons between E-HIV, C-HIV and HC (Fig. 7e). The CD45RA^-^CCR7^+^ T_CM_ phenotype was more frequent amongst CD8^+^CXCR5^+^ T cells compared with CD8^+^CXCR5^-^ T cells, (p = 0.0001), whereas CD8^+^CXCR5^-^ T cells were more likely to be T_EMRA_ (Fig. 7f). CD8^+^CXCR5^+^ T cells more frequently expressed PD-1, median (IQR) 70.33 (60.43–78.50) than CD8^+^CXCR5^−^T cells median (IQR) 27.23 (15.47–35.39) (p < 0.0001) (Fig. 7g). CD8^+^CXCR5^+^ cells were more frequently CD32^+^, median (IQR) 8.56 (5.65–11.31) than CD8^+^CXCR5^−^ cells median (IQR) 4.96 (3.75–6.93), (p < 0.0001) (Fig. 7h). The frequency of CD8^+^CXCR5^+^T_CM_ and CD8^+^CXCR5^−^T_CM_ expressing PD-1 or CD32 did not differ in those with and without HIV infection (Supplementary Fig. S15). The frequency of both CD8^+^CXCR5^+^ and CXCR5^−^ cell subsets positive for CD32 or PD-1 was not significantly altered by vaccination with QIV at Day 7 (Fig. 7i,j and Supplementary Fig. S16).

### Attenuation of cellular activation and lower antibody titres post QIV in those previously vaccinated

**Figure 8:**
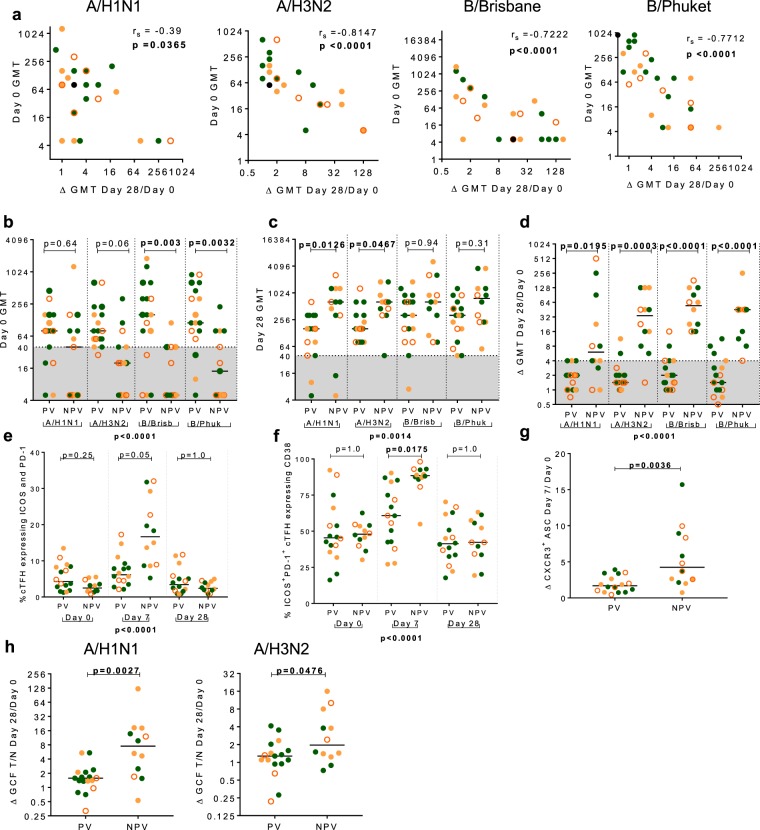
Activation of T and B cells post QIV is attenuated in those self-reporting vaccination with seasonal influenza vaccine in the preceding three years. (**a**) Panels show inverse correlation between Day 0 HAI titre and fold change in HAI titre at Day 28 in A/H1N1, A/H3N2, B/Brisbane and B/Phuket. Comparison of HAI titre in those self-reporting a previous history of vaccination (PV) or no previous vaccination (NPV) at (**b**) baseline and (**c**) Day 28 against A/H1N1, A/H3N2, B/Brisbane and B/Phuket. Shaded area indicates HAI titre <40. (**d**) Comparison in the fold change in HAI titre between PV and NPV against A/H1N1, A/H3N2, B/Brisbane and B/Phuket. Shaded area indicates HAI titre fold change <4. (**e**) Comparison of the fr**e**quency of cT_FH_ that expressed ICOS and PD-1 at all time points in PV and NPV. (**f**) Comparison of the **f**requency of ICOS^+^PD-1^+^ cT_FH_ that expressed CD38^+^ at all time-points in PV and NPV. (**g**) Day 7 fold change in the frequency of CXCR3^+^ ASCs comparing PV and NPV. (h) The fold change in GCF T/N ratio against A/H1N1 and, A/H3N2 comparing PV and those NPV. Lines are drawn at the median. Individual data points are shown on graph panels: HC, filled green circles; E-HIV, filled orange circles; C-HIV, open orange circles. Spearman’s rank correlations coefficient, r_s_ is shown. P values were derived using a Kruskal-Wallis test (p value below graph) and p values of the Dunn’s multiple comparisons test (above graph). Comparisons between two groups were performed using a two-tailed Mann-Whitney U test.

Given the high level of pre-existing seroprotection against influenza in the cohort, the relationship between baseline HAI titre, previous vaccine exposure and cellular and humoral response following QIV was investigated. Unlike activation of cT_FH_ at Day 7, where there was a positive relationship, baseline HAI titre correlated inversely with fold change in HAI titre for all four influenza strains (Fig. 8a and Supplementary Table S2).

Participants were categorized by their self-reported vaccination history in the preceding three years as previously vaccinated (PV), n = 17 (at least one influenza vaccination) or not previously vaccinated (NPV), n = 13 (no influenza vaccination). Thirteen of the seventeen PV (76.5%) had received it in two, or all three, of the previous three years. There was a trend for baseline HAI titre to be lower in those NPV compared with those PV, which was statistically significant for influenza-B strains; B/Brisbane (p = 0.003) and B/Phuket (p = 0.0032), with no differences at Day 7 (Fig. 8b and Supplementary Fig. S17). At Day 28 post QIV, there was a trend for HAI titre to be higher in those NPV compared with those PV, which was significant for influenza-A strains, A/H1N1 (p = 0.0126) and A/H3N2 (p = 0.0467) (Fig. 8c). The Day 28-fold change in HAI titre was significantly higher for those NPV compared with those PV for all four influenza-A and influenza-B strains (p < 0.0001) (Fig. 8d).

Cellular activation post QIV was compared in those PV or NPV. The frequency of PD-1^+^ICOS^+^cT_FH_ was significantly higher in those NPV compared with those PV at Day 7, but not at Day 0 or 28 (p < 0.0001) (Fig. 8e). PD-1^+^ICOS^+^cT_FH_ more frequently expressed CD38 in those NPV, compared with those PV at Day 7, (p < 0.0001) (Fig. 8f). There was no difference in the frequency of cT_FH_ at any time point between those NPV and those PV (Supplementary Fig. S18). The fold change in the frequency of CXCR3^+^ASC at Day 7 was higher in NPV compared with those PV (p = 0.0036) (Fig. 8g and Supplementary Fig. S19). Differences in the frequencies of activated cT_FH_ and activated ASCs at Day 7 post QIV were not significant, for the most part, in those with baseline SP compared with those NSP (Supplementary Figs S20 and S21). Reflecting our findings in the serum, the fold change in GCF antibody T/N ratio was higher in those NPV compared with PV for both A/H1N1 and A/H3N2 (Fig. 8h).

## Discussion

In this cohort of PLWH with suppressed viraemia, we demonstrate functional immune recovery equivalent to control subjects, reflected in humoral and cellular responses to QIV, irrespective of when ART was started. Our findings support the use of QIV in immunization programmes for PLWH and the use of ART to promote recovery of vaccine-induced immunity. Humoral responses in serum and GCF were closely correlated, and were unaffected by suppressed HIV infection. This indicates measurement of influenza-A-specific IgG in GCF is a potential alternative to serum sampling that should prove robust across different patient populations.

Multi-dimensional and two-dimensional analysis of the circulating T cell response revealed distinct CD4^+^CXCR5^+^ T cell subsets that responded differently to immunization but were unaffected by treated and suppressed HIV infection. The frequency of activated CD4^+^CXCR5^+^CXCR3^+^ICOS^+^PD-1^+^CD38^+^ cT_FH_ (P2) increased at Day 7 following QIV in the majority of individuals, and returned to baseline frequency by Day 28 similar to previous reports^38^. A rare subset of circulating CD4^+^CXCR5^+^ T cells that expressed high levels of CD32 (P1), was phenotypically different from cT_FH_, and was not increased in frequency by vaccination with QIV. Expression levels of differentiation and survival makers suggested they were highly differentiated memory cells. A third subset of CD4^+^CXCR5^+^ T cells (P3), whilst sharing phenotypic similarities with cT_FH_, was hierarchically dominant but expressed persistently low levels of cT_FH_ activation markers.

Our data support findings from several studies linking influenza vaccine-induced activation of cT_FH_ with both the ASC response and subsequent measurements of circulating antibody^14,16,38^. The subset that responded to QIV was consistent with descriptions of cT_FH;_ in that they were CD27^mid/hi^CD127^hi^CD28^hi^CD45RA^neg^CCR7^mid^ reflecting a central memory phenotype, bore proteins for cellular survival signals including IL-7R and up-regulated activation markers PD-1, ICOS and CD38 at one week post vaccination. Of significance for vaccine immunogenicity, the frequency of activated cells was associated with the fold change in antibody titre and therefore with subsequent seroprotection. cT_FH_ boost memory to influenza-specific antigen as they induce memory B cells to differentiate into ASCs^14^. CD8^+^ CXCR5^+^ cytotoxic T-Follicular cells circulated at a frequency of 1% of CD8^+^ T cells. They had a similar phenotype to cT_FH_ but were not activated by QIV. Previous work indicates these cells to have potent T-cytotoxic anti-viral properties, and although not activated by QIV, could be a target for future influenza vaccines designed to induce a T cell response^18^.

The cellular response and high seroprotection rates, equivalent to healthcare control subjects, that we observed, was in individuals with fully suppressed HIV infection and good immune recovery (median CD4 count >600 cells/μl). This was regardless of interval from primary infection to treatment initiation and differs from studies reporting inferior responses to seasonal influenza vaccine in PLWH. We recruited a male cohort of PLWH, in their fifth to sixth decade of life and focused on the peak (Day 7) cellular response. Reports of a suboptimal response to trivalent vaccine in PLWH have been in cohorts where generalization to our study may be difficult, for example post-menopausal women, older people or adolescents living with HIV infection^10–12,21,39,40^. One study has indicated that the follicular reaction to influenza vaccine is different in PLWH, however the extent of pre-existing compromise affecting secondary lymphoid tissue from unchecked viraemia prior to starting ART is likely to vary greatly in PLWH^41^. Despite the HC being slightly younger than PLWH, responses in the three groups were equivalent. Although we did not find evidence of a weakened response to QIV in these relatively young men living with HIV infection, we cannot exclude this in older PLWH.

Unlike our findings in treated, suppressed HIV infection, recent previous exposure to seasonal influenza vaccine was associated with significantly attenuated cT_FH_ activation after vaccination with QIV. This was not directly linked to serum HAI titres prior to vaccination, but was associated with lower titres post vaccination. Our data are in accordance with a report investigating HAI titres in healthcare personnel who undergo repeated vaccination^42^. The relationship between pre-existing antibody titre and vaccination response is complex. Some data indicate pre-existing antibody titres can negatively impact the humoral response, although this effect may be reduced in PLWH, paradoxically promoting vaccine immunogenicity^43^. In contrast, data from older people, finding pre-existing influenza-specific immunity is predictive of post vaccination antibody titres, may reflect the spectrum of immune competence in the elderly^44,45^. Our data indicate a role for cT_FH_ in regulating the change in antibody titre with vaccination.

We observed induction of CD32^+^cT_FH_ at Day 7 in t-SNE and 2-D analyses which indicates potential functional significance for this FcγR on CD4^+^ T cells responding to immunisation. FUN-II is the CD32 antibody clone used in this study. It cannot distinguish between the CD32 isotypes CD32a (activating) and CD32b (inhibitory), a limitation of the recent studies using this clone to investigate HIV-DNA in CD4^+^ T cells^26,46^. Determination of which isotype is being expressed on the CD4^+^ T cells we describe should be undertaken to indicate its mechanistic significance.

We have used different analysis modalities to explore this complex dataset; each has limitations. These include inability to identify rare populations using two-dimensional analysis, inability to infer hierarchy using t-SNE and potential for natural biological variability in rare cell populations to affect clustering analysis in SPADE over time. Through a combination of these approaches we were able to tease out the specific T cell subset responding to QIV, adding to the growing body of work indicating cT_FH_ are operational in vaccine-induced immunity. Our data identify a specific subset of cT_FH_ that have an important function for both inducing seroprotection and controlling the antibody response and indicate that this can function optimally where HIV infection is treated and suppressed.

## Materials and Methods

### Study enrollment

Participants were enrolled into the study during the 2017–18 Northern Hemisphere influenza season, from September 2017 until February 2018. All participants gave written, informed consent prior to enrolment. Healthcare control subjects (HC) (n = 14) receiving influenza vaccine as part of the yearly occupational health initiative, were enrolled from the staff of Imperial College London and Imperial College Healthcare NHS Trust, London UK. Male people living with HIV infection (PLWH) (n = 16), receiving influenza vaccine as part of routine care and treated for early HIV infection (E-HIV) or chronic HIV infection (C-HIV) were enrolled from the Jefferiss Wing Clinic, Imperial College Healthcare Trust, London UK. Individuals diagnosed with primary HIV infection who started ART within three months of diagnosis (E-HIV), (n = 8) were identified from a database of individuals attending a study of ART treatment of primary HIV infection (HEATHER study). Individuals attending the routine HIV outpatients clinic were classified as, HIV infection treated during the chronic phase (C-HIV), (n = 8). Participants were asked to provide details concerning their general health and lifestyle and about their influenza vaccination history over the previous three influenza seasons prior to enrolment in the study. Eleven HC (78.6%), reported a negative HIV test result within the previous 3.5 years. In these participants, using our standard flow cytometry method, the CD4 percentage of total lymphocytes was median (IQR) 30.27 (24.25–35.94). All those with HIV infection were taking ART and had an undetectable viral load (<50 RNA copies/ml) at the time of sampling, and for a minimum of six months prior to inclusion in the study. Individuals were classified as having evidence of seroprotection (SP) against each antigen if the HAI titre was ≥40.

The study was approved by the London-Brighton & Sussex Research Ethics Committee, (REC ref 17/LO/1311)and performed to Good Clinical Practice guidelines in accordance with the UK Policy Framework for Health and Social Care Research. All participants provided written informed consent prior to enrolment.

### Unsupervised computer algorithms

FACS data suitable for machine learning analysis was available for 23 participants. t-SNE was performed using a FlowJo v10.4.2 plug-in. Data from all time points were gated for the sub-population desired (CD3^+^CD4^+^ or CD3^+^CD8^+^ or CD19^+^) by running three individual analyses. The t-SNEs were run with 1000 iterations, a perplexity of 20, Eta (learning rate) of 200 and a Theta of 0.5.

Spanning-tree Progression Analysis of Density-normalized Events (SPADE) analysis was performed using FCS express v6+ Research Edition. Data from all time points was gated for the sub-population desired CD3^+^CD4^+^. SPADE was run using the transformation SPADE function in FCS express with parameters of 30 clusters, maximum iterations 100, and minimum percent cells per cluster of 0.1. The parameters used in the transformation were CD8, CD4, PD-1, ICOS, CXCR3, CD45RA, CD32, CXCR5, CCR7 and CD28. The resulting output for both t-SNE and SPADE were plotted on a heatmaps and analysed for relative expression of the parameters included.

### Statistical analysis

All statistical analyses were performed using GraphPad Prism software, v7.04. Spearman’s rank correlation coefficient (r_s_) was calculated to compare the relationship between two independent continuous variables. Comparisons between two categorical parameters were performed using a two tailed Mann-Whitney U test. Comparisons between multiple groups were performed using a Kruskal-Wallis test and Dunn’s multiple comparison test. Multiple intra-individual comparisons between time-points were performed using a Friedman test and Dunn’s multiple comparisons test. For all statistical analysis, p values of less than 0.05 were considered significant and non-significant values were rounded to two significant figures. Demographics were analysed using SPSS where the percentiles were calculated using weighted average where median was defined as 50^th^ percentile, upper IQ as 75th and lower as 25th. Missing data was at random and was dealt with by listwise deletion for each timepoint. Two participants had missing or incomplete time-courses to perform comparative cellular experiments and were therefore excluded from analysis of cellular events. One participant had no serum available for analysis at Day 28 but data were available from the first two time points.

## Supplementary information

Supplementary methods and data

## Data Availability

All data referred to are available in the manuscript and its supplementary documents.
