# Supplementary material for: Responses to Quadrivalent Influenza Vaccine Reveal Distinct Circulating CD4+CXCR5+ T Cell Subsets in Men Living with HIV
Source: Sci Rep. 2019 Oct 30;9:15650. doi: 10.1038/s41598-019-51961-9 (PMC6821795; doi:10.1038/s41598-019-51961-9)
Supplement: Supplementary file 1 — Supplementary methods and data [file 41598_2019_51961_MOESM1_ESM.docx]

Title: RESPONSES TO QUADRIVALENT INFLUENZA VACCINE REVEAL DISTINCT CIRCULATING CD4+CXCR5+ T-CELL SUBSETS IN MEN LIVING WITH HIV

**Authors:** Megan E. Cole^1^, Zainab Saeed^1^, A. Torm Shaw^1^, Yanping Guo^2^, Katja Höschler^3^, Alan Winston^1,4^, Graham S. Cooke^1^, Sarah Fidler^1,4^, Graham P. Taylor^1,5^, and Katrina M. Pollock^1^*

**Affiliations:**

^1^Section of Clinical Virology, Department of Infectious Disease, Imperial College London, London, UK.

^2^St. Mary’s FACS facility, Imperial College London, London, UK.

^3^Respiratory Virus Unit, Virus Reference Department, National Infections Service, Public Health England, UK.

^4^Clinical Trials Centre, Jefferiss Wing, Imperial College Healthcare NHS Trust, London, UK.

^5^National Centre for Human Retrovirology, Imperial College Healthcare NHS Trust, London, UK

*To whom correspondence should be addressed: Dr Katrina M Pollock, Section of Clinical Virology, Department of Infectious Disease, Imperial College London, Rm 455, Medical School, Norfolk Place, London, UK, W2 1PG.

Email: k.pollock@imperial.ac.uk

**Processing of sera, oral fluid and tissue**

Sera, oral fluid and peripheral blood mononuclear cells were processed according to standard laboratory practice and stored either viably cryopreserved at -150^o^C or at -20 or -80^o^C. PBMC were extracted using a Histopaque gradient, cells were washed and stored at -150^o^C until further analysis. Serum and Plasma were extracted post separation via centrifugation, aliquot and stored at -80 ^o^C until further analysis.

**Clinical data collection**

Clinical and demographic data from participants, were mined from electronic patient records following provision of written informed consent. Absolute cell numbers performed by the Clinical Pathology Laboratory were available for PLWH using the Navios tetra system (Beckman Coulter Life Sciences) to report the absolute number of CD3^+^, CD4^+^ and CD8^+^ cells per μl of whole blood.

**Hemagglutination inhibition assay**

Paired sera were analysed to ISO 15189 standards by Hemagglutination Inhibition with the following viruses, A/Michigan/45/2015(H1N1)pdm09, A/Hong Kong/4801/2014(H3N2) and B/Brisbane/60/08 & B/Phuket/3073/2013. Sera were prepared for the assay to eliminate non-specific inhibitors and anti-species Has. HAI was run by Public Health England using methods previously described.

**Detection of Influenza-A specific IgG in gingival crevicular fluid**

Paired oral fluid samples from Day 0 and Day 28 were analysed by indirect ELISA using two antigens; HA1 fraction of the HA; A/California/7/2009 (H1N1pdm09) and A/Hong Kong/4801/2014 (H3N2-like virus). Assays were performed using a pre-validated control human oral fluid. The test to negative (T/N) ratio for each oral fluid with each antigen was calculated by dividing the OD450 with the OD450 of a negative control. Samples with T/N ratio < 2 were unreactive. The assay has been developed by Public Health England for use with GCF samples collected using Oracol® sponges.

**Flow Cytometry**

Flow cytometry was performed on thawed viably cryopreserved peripheral blood mononuclear cells (PBMCs), stained with Panel 1 (Table S3), fixed using 4% paraformaldehyde solution (BD biosences) and acquired immediately on BD LSR Fortessa. To facilitate the study of rare T-cell subsets, all events were collected; median (IQR) 4.0 x 10^6^ (3.1x 10^6^–4.8x10^6^) events per sample. All flow cytometry data were analysed in FlowJo 10.4.2 (FlowJo, LLC).

**Analysis of multi parameter FACS data using unsupervised computer algorithms**

FACS data suitable for machine learning analysis was available for 23 participants. t-SNE was performed using a FlowJo v10.4.2 plug-in. Data from all time points was gated for the sub-population desired (CD3^+^CD4^+^ or CD3^+^CD8^+^ or CD19^+^). Data was condensed using down-sampling (5000 events), and combined using the FlowJo concatenate function. Files were concatenated sequentially in participant then time point order. To compare outputs for each individual at each time point, the concatenated files were viewed as SAMPLE ID versus forward scatter and the SAMPLE ID from the Flow Jo file used to identify FV1, FV2 and FV3 time points, which were sequentially gated and labelled. Using the t-SNE function, three individual analyses were run defined by cell lineage markers; live CD3^+^CD4^+^ cells, live CD3^+^CD8^+^ cells and live CD19^+^ cells.

Spanning-tree Progression Analysis of Density-normalized Events (SPADE) analysis was performed using FCS express v6+ Research Edition. The combined data at all-time points from 23 of the participants was gated on live CD3^+^ cells and the data was condensed using down-sampling (5000 events), then combined using the concatenate function in FlowJo. The concatenated file was imported into FCS express and CD4^+^ T-cells were selected. SPADE was run using the transformation SPADE function in FCS express 6.
